# Isopathy in the Dominant School

**Published:** 1893-11

**Authors:** 


### HOMŒOPATHIC PHYSICIAN,

A MONTHLY JOURNAL OF

HOMŒOPATHIC MATERIA MEDICA AND CLINICAL MEDICINE.

"If our school ever gives up the strict inductive method of Hahnemann, we are lost, and deserve only to be mentioned as a caricature in the history of medicine."—CONSTANTINE HERING.

Vol. XIII. NOVEMBER, 1893. No. 11.

### EDITORIAL.

ISOPATHY IN THE DOMINANT SCHOOL.—It has been said in previous editorials in this journal that by the slow process of evolution the practitioners of the old school of medicine were certainly approaching the tenets of Homœopathy. A careful scanning of the rich literature in the best journals of the "regular" school will convince any one of the truth of this assertion.

At present much attention is being paid to Isopathy, or as they prefer to call it, "Organopathy." The essential nature of this practice is not the giving of morbific products to cure disease, but the administration of juices extracted from healthy glands or other tissues to cure diseases occurring in the corresponding glands or tissues.

Dr. Wm. A. Hammond, retired Surgeon-General of the United States Army, has delivered a lecture advocating Organopathy. He reports excellent results from the administration of Cerebrine, extracted from the healthy brain of the sheep in the treatment of neurasthenia, hysteria, paralysis, and insomnia.

Professor C. L. Dana, of New York, has been using extract of the thyroid gland and of the brain tissue as well as rabic virus, and tuberculin in the treatment of paralysis, progressive muscular atrophy, epilepsy, and tubercular tumor of the brain.

Dr. Constantin Paul, of the Charité Hospital, Paris, has been using extract of gray matter of the brain of the sheep for the treatment of simple neurasthenia, neurasthenia with chlorosis, and locomotor ataxia.

Dr. Cullerre, of the lunatic asylum at Roche-sur-Yon, has been using similar substances in the treatment of the insane.

A fuller account than is here given of this new practice can be found in an article in *The New York Medical Times* for October, 1893, page 199.

It would appear from the statements made in the article referred to that there is the usual proportion of success to failure that attends the use of new remedies. Of course, these preparations are administered almost entirely empirically, and so there is nothing surprising in the results.

No suggestion for the testing of these drugs upon the healthy that some idea of their scope and sphere of action could be formed is offered, except by Dr. Hammond, who does make some crude tests of this kind, and reports the symptoms obtained. Unfortunately, these symptoms are not of a character to enable any one to make an intelligent prescription of the substance that has been thus tested upon the healthy, consequently there is no escape from the empirical method of prescribing.

Although this Isopathy or Organopathy is a long way off from the plain, straight, and satisfactory method of the law of similars, still it represents progress. Compare it for a moment with the old methods of bleeding, purging, salivation, and like devices, and it represents an enormous advance. Compared even with the usual tactics now prevalent of massive doses of Quinine and like drugs on so-called rational principles, it is a great advance. We may look forward in the not very distant future to a proposition being made by some prominent light of old medicine for a systematic proving of these substances upon the healthy and the administering of them on the line of the "suggestions" found in the provings. Hence, we may all live to hear of "suggestive medicine" as a thin disguise for practice according to the maxims of Homœopathy, but without the need of using that hated name, or giving its founder and its votaries the credit due them.

The lesson to be derived from a comtemplation of these changes going on in the old school is the inspiration it must give to professed homœopathists to hold with a more tenacious grasp the powerful instrument for good which they now possess in the law of the similars. To the lukewarm brethren in the faith of pure Homœopathy, many of whom read this journal, this lesson is particularly applicable. For them these lines are written, as are most of these editorials which appear in these pages from month to month. To them the appeal is herewith made to look more closely into the new-school principles, to carefully study its materia medica, to fearlessly apply the similar remedy, the single proved drug in the minimum dose, and trustfully await the favorable result. To avoid all adjuvant methods of prescribing which are the outgrowth of old-school experience, confident in the expectation that sooner or later they will be abandoned by the old-school originators themselves as they come nearer and nearer our methods of healing the sick.

